# Phosphorylation Signals Downstream of Dopamine Receptors in Emotional Behaviors: Association with Preference and Avoidance

**DOI:** 10.3390/ijms231911643

**Published:** 2022-10-01

**Authors:** Xinjian Zhang, Daisuke Tsuboi, Yasuhiro Funahashi, Yukie Yamahashi, Kozo Kaibuchi, Taku Nagai

**Affiliations:** 1 Division of Behavioral Neuropharmacology, International Center for Brain Science (ICBS), Fujita Health University, Toyoake 470-1192, Aichi, Japan; 2 Center for Medical Science, Fujita Health University, Toyoake 470-1192, Aichi, Japan; 3 Division of Cell Biology, International Center for Brain Science (ICBS), Fujita Health University, Toyoake 470-1192, Aichi, Japan

**Keywords:** dopamine, dopamine receptors, nucleus accumbens, phosphorylation signals, rewarding behavior, aversive behavior

## Abstract

Dopamine regulates emotional behaviors, including rewarding and aversive behaviors, through the mesolimbic dopaminergic pathway, which projects dopamine neurons from the ventral tegmental area to the nucleus accumbens (NAc). Protein phosphorylation is critical for intracellular signaling pathways and physiological functions, which are regulated by neurotransmitters in the brain. Previous studies have demonstrated that dopamine stimulated the phosphorylation of intracellular substrates, such as receptors, ion channels, and transcription factors, to regulate neuronal excitability and synaptic plasticity through dopamine receptors. We also established a novel database called KANPHOS that provides information on phosphorylation signals downstream of monoamines identified by our kinase substrate screening methods, including dopamine, in addition to those reported in the literature. Recent advances in proteomics techniques have enabled us to clarify the mechanisms through which dopamine controls rewarding and aversive behaviors through signal pathways in the NAc. In this review, we discuss the intracellular phosphorylation signals regulated by dopamine in these two emotional behaviors.

## 1. Introduction

Dopamine was discovered as a neurotransmitter in the brain by Dr. Arvid Carlsson in 1957 [1]. It plays important roles in a number of brain functions, including motor function, motivation, learning, and rewards [2,3,4,5]. Dysfunctions in the dopaminergic system underlie various neuropsychological diseases, including Parkinson’s disease, schizophrenia, drug addiction, attention deficit hyperactivity disorder (ADHD), post-traumatic stress disorder, major depression, and restless legs syndrome [6,7,8,9,10,11,12,13].

There are several dopaminergic pathways in the brain. Two major pathways are the nigrostriatal pathway and mesolimbic pathway, which project dopamine neurons from the substantia nigra to the dorsal striatum, and from the ventral tegmental area (VTA) to the ventral striatum, including the nucleus accumbens (NAc). Nigrostriatal dopamine neurons play an important role in motor functions, and their dysfunction causes Parkinson’s disease [6]. Accumulated evidence has revealed that dopamine transmission from the VTA to NAc is critical for controlling emotional behaviors, including rewarding and aversive behaviors [14,15]. Emotional behaviors contribute to the better survival of organisms. For example, learning and memory related to rewards and aversion allow animals to efficiently obtain food or to escape from danger, in line with the theory of “survival of the fittest”.

Classical conditioning is a kind of simple reward-associated learning [16]. Extracellular dopamine levels increase in the NAc when a reward or reward cue is delivered [17]. Cue-evoked dopamine is essential for reward-associative learning, as demonstrated by both the pharmacological inhibition of dopamine receptors [17] and the optogenetic inhibition of VTA dopamine neurons [18]. Cue-evoked dopamine also promotes conditioned responses during learning [19].

In contrast with the phasic firing response of VTA dopamine neurons to rewarding stimuli, their reactions to aversive stimuli are not homologous. The firing of some VTA dopamine neurons is activated, whereas the others are transiently suppressed. The inactivation of VTA dopamine neurons elicited by the optogenetic inhibition of dopamine neurons [20] or the activation of VTA GABAergic interneurons [21] has previously been shown to induce aversive behavior. Aversive behavior induced by the inactivation of VTA dopamine neurons is mediated by dopamine D2 receptors in the NAc [20]. Therefore, reductions in dopaminergic activity are associated with aversive behavior.

Dopamine exerts its function through dopamine receptors, which are a class of G-protein-coupled receptors. There are five types of dopamine receptors: D1–5. D1 and D5 are coupled to Gs, the stimulation of which increases the intracellular concentration of the second messenger cyclic adenosine monophosphate (cAMP) through adenylate cyclase (AC), thereby activating cAMP-dependent protein kinase (PKA). On the other hand, D2, D3, and D4 are coupled to Gi, the activation of which results in the inhibition of the cAMP/PKA signaling pathway [22]. Therefore, D1 and D5 receptors are called the D1-like receptor family. D2, D3, and D4 receptors are called the D2-like receptor family. In addition, D2 receptors are known to transduce the signal via noncanonical G protein–independent interactions with β-arrestins [23]. Arrestin recruitment to D2R has been shown to mediate an antipsychotic effect in several studies [24], and to mediate locomotion, but not incentive motivation, in another study [25]. In this review, we focused on the canonical phosphorylation signaling pathway. Recent advances in proteomics techniques have enabled us to clarify the novel mechanisms through which dopamine controls both rewarding and aversive behaviors through protein phosphorylation in the NAc. In this review, we discuss the intracellular phosphorylation signals regulated by dopamine in these two emotional behaviors.

## 2. Functional Model for Dopamine D1 Receptors Expressing Medium Spiny Neurons (D1R-MSN) and Dopamine D2 Receptors Expressing Medium Spiny Neurons (D2R-MSN) in Rewarding and Aversive Behaviors

**Figure 1:**
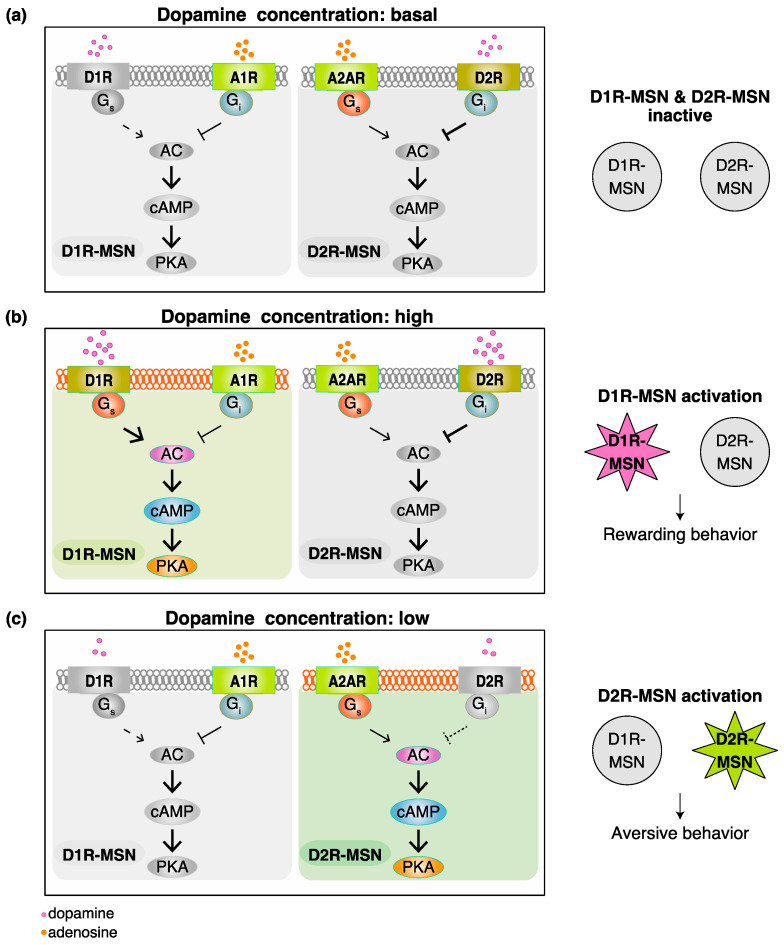
Dopamine concentrations function as a switch in the active state shift between D1R-MSN and D2R-MSN to regulate rewarding and aversive behaviors, respectively. (**a**) At the concentration of dopamine under basal conditions, D1R-MSN and D2R-MSN are both inactive. (**b**) When the concentration of dopamine is high, D1R-MSN is activated, consequently leading to rewarding behavior. Under pathophysiological conditions, the dopamine hyperfunctional state may be associated with schizophrenia and drug addiction. (**c**) When the concentration of dopamine is low, D2R-MSN is activated to induce aversive behavior. Under pathophysiological conditions, the dopamine hypofunctional state may be associated with Parkinson’s disease, attention deficit hyperactivity disorder, and restless legs syndrome. D1R, dopamine D1 receptor; D2R, dopamine D2 receptor; A1R, adenosine A1 receptor; A2AR, adenosine A2A receptor; MSN, medium spiny neuron; Gs, stimulatory G protein; Gi, inhibitory G protein; AC, adenylate cyclase; cAMP, cyclic adenosine monophosphate; PKA, protein kinase A.

In the NAc, approximately 95% of neurons are medium spiny neurons (MSN) receiving dopaminergic regulation from the VTA [26,27]. There are two types of MSN—dopamine D1 receptors expressing D1R-MSN and dopamine D2 receptors expressing D2R-MSN. Dr. Hikida and co-workers expressed the tetanus toxin light chain, which is a bacterial toxin that cleaves the synaptic-vesicle-associated VAMP2 protein and abolishes neurotransmitter release from synaptic vesicles, in D1R-MSN and D2R-MSN [28]. The cell-type-specific inhibition of D1R-MSN with the tetanus toxin resulted in diminished rewarding behavior, while that of D2R-MSN led to diminished aversive behavior. These findings indicate that the activation of D1R-MSN regulates rewarding behavior, while that of D2R-MSN encodes aversive behavior. Striatal tonic dopamine release sustains basal extracellular dopamine concentrations in the NAc. Rewarding stimuli may induce phasic dopamine release to increase dopamine concentrations, and aversive stimuli inhibit tonic dopamine release to decrease dopamine concentrations [15,29]. Although dopamine was previously regarded as a volume transmitter that signals slowly and inaccurately, a recent study revealed that striatal dopamine secretion was mediated by sparse active-zone-like release sites supporting the dynamic function of dopamine [30,31]. According to the nature of D1R and D2R, a model has been proposed to explain how the active state shifts between D1R-MSN and D2R-MSN, regulating rewarding and aversive behaviors, respectively (Figure 1).

D1R is coupled to Gs, the stimulation of which activates PKA to trigger D1R-MSN. On the other hand, D2R is coupled to Gi, the activation of which results in the inhibition of the cAMP/PKA signaling pathway and, in turn, the suppression of D2R-MSN. D2R has a markedly higher affinity for dopamine than D1R [32], and the Kd values of D1R and D2R are around 200 nM and 10 nM, respectively [33]. Therefore, basal dopamine concentrations, which have been reported to be around 10 nM [34], are insufficient to activate D1R and induce the activation of D1R-MSN, but are adequate to activate D2R and suppress D2R-MSN activity (Figure 1a). Rewarding stimuli markedly increase extracellular dopamine (e.g., cocaine intake has been shown to increase dopamine levels to more than 280 nM [35]), which is followed by the activation of both D1R and D2R. The stimulation of D1R induces the activation of D1R-MSN, whereas that of D2R suppresses D2R-MSN activity (Figure 1b). Aversive stimuli reduce extracellular dopamine to levels (e.g., electric footshock has decreased dopamine levels to less than 10 nM [36]) at which neither D1R nor D2R is activated. Furthermore, D1R-MSN expresses the adenosine A1 receptor (A1R) [37], which couples to the Gi protein, and D2R-MSN expresses the adenosine A2A receptor (A2AR) [37], which couples to the Gs protein. Basal extracellular adenosine concentrations are sufficient to tonically activate A1R and A2AR [38]. Therefore, D2R-MSN is activated due to the disinhibition in the D2R signal (Figure 1c). With the cooperation of adenosine, dopamine concentrations function as a switch for controlling the active state shift between D1R-MSN and D2R-MSN, thereby regulating rewarding and aversive behaviors, respectively [39]. A recent study showed dopamine terminals in the ventral NAc medial shell are excited, but other NAc regions are inhibited by aversive stimuli [40]. Although changes in the dopaminergic activity seem to be different in the subregion of NAc after aversive stimuli, this model may be applicable for NAc regions, except for the ventral medial shell.

## 3. PKA and PKA Substrates

Rewarding and aversive behaviors in rodents are monitored by conditioned place preference (CPP) and passive avoidance tests, respectively. The inhibition of PKA in the NAc blocks amphetamine-induced place preference [41] and its activation in D1R-MSN enhances cocaine-induced place preference [42]. The inhibition of PKA in D2R-MSN blocks foot shock-produced avoidance, whereas its activation increases foot shock-produced avoidance [43]. These findings strongly demonstrate that PKA plays an important role in D1R-MSN and D2R-MSN to regulate rewarding and aversive behaviors, respectively.

**Figure 2:**
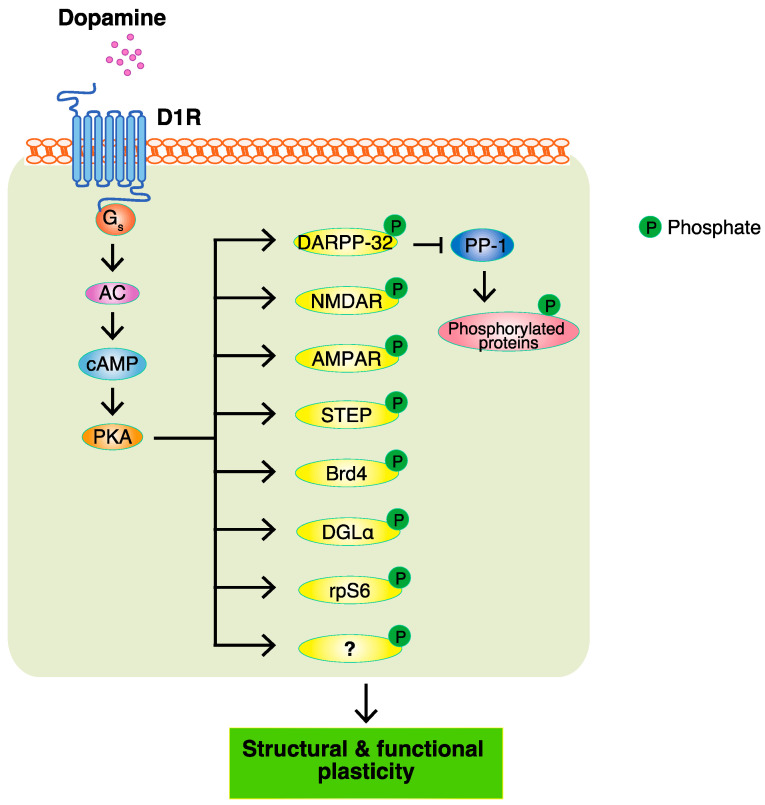
The PKA-dependent phosphorylation of receptors, ion channels, transcription factors, and other proteins accounts for structural and functional plasticity regulated by dopamine. D1R, dopamine D1 receptor; Gs, stimulatory G protein; AC, adenylate cyclase; cAMP, cyclic adenosine monophosphate; PKA, protein kinase A; DARPP-32, dopamine- and cAMP-regulated phosphoprotein of 32 kDa; PP1, protein phosphatase-1; NMDAR, NMDA receptor; AMPAR, AMPA receptor; STEP, striatal-enriched protein tyrosine phosphatase; Brd4, bromodomain-containing protein 4; DGLα, diacylglycerol lipase-α; rpS6, ribosomal protein S6. “?” indicates unknown PKA substrates.

The PKA-dependent phosphorylation of receptors, ion channels, transcription factors, and other proteins accounts for the structural and functional plasticity regulated by dopamine [44] (Figure 2). Dr. Paul Greengard and colleagues unlocked a new world for intracellular dopamine signaling research. They performed a systematic survey of cAMP-activated phosphoproteins in various brain regions and found that PKA phosphorylated dopamine- and cAMP-regulated phosphoprotein of 32 kDa (DARPP-32), which is highly expressed in the striatum, including the NAc [45]. Phosphorylated DARPP-32 at Thr34 by PKA inhibits the activity of protein phosphatase-1 (PP1). The inhibition of dephosphorylation results in the accumulation of phosphorylated proteins. In addition to DARPP-32, inhibitor-1 is an endogenous inhibitor of PP-1. Knockout mice lacking one or both of these PP-1 inhibitors exhibited a reduction in the rewarding effects of cocaine in the CPP test [46]. Therefore, DARPP-32 promotes the function of dopamine in emotional behaviors by suppressing protein dephosphorylation.

AMPA-type and NMDA-type glutamate receptors are abundantly expressed in the striatum, which is involved in synaptic plasticity [47,48]. The phosphorylation of the AMPA receptor subunit GluA1 at Ser845 by PKA has been shown to enhance the surface expression of AMPA receptors [49]. AMPAR knock-out mice lacking GluA1 or GluA2 have been shown to disrupt the stimulus-reward learning or food-induced place preference [50,51]. PKA has also been shown to enhance the surface expression of NMDA receptors by phosphorylating striatal-enriched protein tyrosine phosphatase (STEP) [52]. NMDA receptors are trafficked to the membrane in a tyrosine phosphorylation-dependent manner, and receptor endocytosis is enhanced by tyrosine phosphatase. STEP has been shown to dephosphorylate the NMDA receptor subunit NR2B at Tyr1472, which is correlated with receptor endocytosis [53]. PKA phosphorylated STEP at Ser221 and Ser49 to decrease the activity of STEP by reducing its affinity for its substrate, thereby sustaining surface NMDA receptor expression [52]. Furthermore, the inactivation of the NMDA receptor in D1R-expressing neurons blocked cocaine-induced place preference [54]. Therefore, dopamine regulates synaptic plasticity through AMPA-type and NMDA-type glutamate receptors to exert its function in emotional behaviors.

Cyclic AMP response element (CRE) binding protein (CREB) is a transcription factor that is activated by the phosphorylation of Ser133, and it mediates the expression of genes that shape the structure and function of neurons [55,56,57]. Although PKA directly phosphorylated CREB Ser133 [58], Dudman et al. demonstrated that D1-receptor-mediated CREB phosphorylation occurred through the PKA-NMDA receptor-CaMK pathway in MSN [59]. PKA phosphorylates NMDA at Ser897 on the NR1 subunit to facilitate NMDA channel activity. The activation of CaMK through increases in cytosolic Ca^2+^ is involved in the phosphorylation of CREB. They also showed that cross-talk between PKA and Ca^2+^ accounted for complex intracellular signaling. Furthermore, CREB activity in D1R-expressing neurons is shown to regulate cocaine-induced place preference [60], indicating that CREB-dependent transcriptional regulation plays a role in the rewarding effect of cocaine.

Besides CREB, dopamine receptors control gene expression through numerous transcription factors [61,62,63] and chromatin regulators [64,65,66,67,68,69]. Bromodomain and extra-terminal (BET) family proteins play a crucial role in regulating gene transcription through bromodomains that specifically bind acetylated lysine residues in histones. Bromodomain-containing protein 4 (Brd4) is a member of the BET family that is activated through its phosphorylation-dependent interaction domain (PDID). Casein kinases have been shown to phosphorylate PDID, which induces a conformational change that unmasks bromodomains, thereby facilitating chromatin binding [69]. PKA also phosphorylated Brd4 PDID in vitro [70], and regulated the recruitment of Brd4 to chromatin in cardiomyocytes [71] and pancreatic β cells [72]. Jones-Tabah et al. recently demonstrated that the inhibition of BET proteins by the bromodomain inhibitor JQ1 suppressed the D1R-induced up-regulation of the gene expression by ~25% [73]. Furthermore, cAMP/PKA signaling promoted the recruitment of Brd4 to dopamine-induced genes in striatal neurons, and the knockdown of Brd4 attenuated D1R-induced gene expression. The systemic and intra-accumbal inhibition of Brd4 with JQ1 attenuated the rewarding effects of cocaine or amphetamine-induced place preference [74,75]. Therefore, these findings indicate that PKA directly or indirectly phosphorylates CREB or Brd4 to promote the gene expression for synaptic plasticity regulated by dopamine.

The endogenous cannabinoid system has been implicated in reward seeking. Endogenous cannabinoids mediate long-term depression and evoke drug (cocaine and ethanol) and natural reward (saccharin)-seeking behaviors [76]. 2-Arachidonoyl glycerol (2-AG) is the most abundant brain endogenous cannabinoid and is primarily synthesized by diacylglycerol lipase-α (DGLα). A recent study reported that PKA directly phosphorylated DGLα at Ser798 in vitro and increased DGLα activity [77]. D1R signaling enhanced the DGLα- and 2-AG-dependent form of synaptic depression in mouse striatal slices; however, this was blocked by the post-synaptic inhibition of PKA. These findings demonstrate that D1R/PKA signaling in D1R-MSN crosstalk with synaptic endocannabinoid signaling may also contribute to the regulation of rewarding behavior.

Ribosomal protein S6 (rpS6) is a component of the small 40S ribosomal subunit implicated in mRNA decoding [78]. rpS6 is phosphorylated at multiple sites by kinases including p70 ribosomal protein S6 kinases 1 and 2 (p70S6K1/2) [79], MAPK [80], PKC [81], and PKA [81,82,83]. D-amphetamine, which increases extracellular dopamine levels, was found to markedly enhance the phosphorylation of rpS6 at the Ser235/236 sites selectively in D1R-MSN through PKA, but not p70S6K1/2, MAPK, or PKC [72]. Increased PKA-dependent rpS6 phosphorylation in striatal MSN did not correlate with changes in the global protein synthesis or the translation of mRNA containing a 5’ terminal oligopyrimidine tract [84]. Therefore, the significance of PKA-dependent rpS6 phosphorylation in D1R-MSN or D2R-MSN needs to be elucidated in more detail.

## 4. Phosphoproteomic Analyses Revealed New Phosphorylation Signals Downstream of Dopamine Receptors in Emotional Behaviors

Major efforts have been made to identify the target substrates of PKA so as to understand the modes of action of dopamine, and a few of its substrates, including DARPP-32, GluA1, and NR1, have been reported. The PKA-mediated phosphorylation of DARPP-32 indirectly accumulates phosphoproteins through the inhibition of protein phosphatase PP1. However, it remains unclear about the proteins directly phosphorylated by PKA. To identify the PKA substrates that are responsible for the function of dopamine, a Kinase-Oriented Substrate Screening (KiOSS) method, a novel comprehensive phosphoproteomic analysis, has been developed. More than 100 novel PKA substrates downstream of dopamine were identified, including Rasgrp2 and Rap1gap [43,85]. Rasgrp2 and Rap1gap are positive and negative regulators, respectively, of Rap1. PKA phosphorylates Rasgrp2 at Ser116, Ser117, Ser554, and Ser586 to increase its activity to Rap1. The phosphorylation of Rap1gap at Ser563 deceased its activity to Rap1 [86]. Therefore, Rap1 is efficiently activated through these two pathways to increase the neuronal excitability of D1R-MSN and to enhance rewarding behavior. The activation of Rap1 in D1R-MSN enhances cocaine-induced place preference, which is blocked in accumbal D1R-MSN-specific Rap1-knockout mice. PKA/Rap1 signaling has also been shown to play an important role in the regulation of aversive behavior by D2R-MSN [43]. The inhibition of PKA/Rap1 signaling in accumbal D2R-MSN impaired aversive behavior in passive avoidance tests, whereas its activation potentiated aversive behavior. Therefore, the PKA/Rap1 pathway is a novel signal downstream of dopamine that is involved in emotional behaviors.

A previous study demonstrated that Rap1 activated B-raf, resulting in the activation of MAP2K, followed by MAPK1/3 [87]. The activation of MAP2K in D1R-MSN potentiated the neuronal excitability of D1R-MSN and cocaine-induced place preference, whereas its inactivation blocked neuronal excitability and cocaine-induced place preference [42]. MAP2K is essential for PKA/Rap1 to enhance cocaine-induced place preference and it has been shown to rescue the deficit in cocaine-induced place preference in accumbal Rap1-knockout mice. These findings indicate that PKA/Rap1 mediates the activation of MAPK1/3 downstream of D1Rs to regulate rewarding behavior. MAPK signaling in accumbal D2R-MSN is also critical for mediating aversive behavior. The inhibition of MAPK signaling in accumbal D2R-MSN by transfecting the dominant negative mutant MAP2K1 reduced foot shock-produced avoidance, whereas its activation by transfecting a constitutively active mutant MAP2K1 enhanced foot shock-produced avoidance [43].

K+ channels of the Kcnq/Kv7 family are proteins that span the cell membrane and, when open, allow the outward flow of K+ ions across the membrane [88]. In the central nervous system, Kcnq channels are mainly composed of Kcnq2 and Kcnq3 subunits. MAPK downstream of D1R/PKA/Rap1 phosphorylates Kcnq2 at Ser414 and Ser476 to negatively regulate its channel activity and increase D1R-MSN membrane excitability. The conditional deletion of Kcnq2 in D1R-MSN enhanced neuronal excitability and cocaine-induced rewarding behavior [89]. These effects were restored by the viral-mediated expression of Kcnq2 in the NAc of Kcnq2 conditional knockout mice, but not phospho-deficient Kcnq2. These findings demonstrated that D1R/PKA/Rap1/MAPK signaling enhanced D1R-MSN excitability through Kcnq2 phosphorylation to regulate rewarding behavior. The phosphorylation of Kcnq2 also plays an important role in aversive behavior. Kcnq2 Thr217 was phosphorylated by PKC downstream of acetylcholine/muscarinic M1 receptor (M1R) signaling. Furthermore, the conditional deletion of Kcnq2 in the NAc enhanced electric foot shock-induced aversive behavior [90]. Therefore, Kcnq2 phosphorylation accounts for dopamine-induced MSN excitability involved in regulating emotional behaviors.

To further identify the MAPK substrates involved in regulating the expression of the genes that account for dopamine functions, Funahashi et al. performed a proteomic analysis using affinity beads coated with CREB-binding protein (CBP), a transcriptional coactivator associated with rewarding behavior [91]. More than 400 CBP-interacting proteins were identified, including Neuronal Per Arnt Sim domain protein 4 (Npas4) and megakaryoblastic leukemia-2 (MKL2). Npas4 plays a role in the expression of activity-dependent genes, such as brain-derived neurotrophic factor (BDNF) and c-Fos, to control synaptic plasticity [92]. MAPK phosphorylates Npas4 at Thr423, Thr427, Ser577, Ser580, Thr611, and Ser615 sites in D1R-MSN, and increases the interaction between Npas4 and CBP, thereby regulating transcriptional activity to enhance reward-related learning and memory. MKL2 is a coactivator of the transcription factor SRF [93], which is essential for cocaine-mediated spine morphogenesis in the NAc and rewarding behavior [94,95]. MAPK has been shown to phosphorylate MKL2 at Ser913, which induced the nuclear localization of MKL2 and increased SRF-dependent transcriptional activity in the neurons [96]. The phosphoproteomic analysis is a powerful tool for identifying new phosphorylation signals downstream of dopamine receptors in emotional behaviors.

## 5. Novel Database of Kinase-Associated Neural Protein Phosphorylation in the Brain: Kinase-Associated Neural PHOspho-Signaling (KANPHOS)

As described above, protein phosphorylation plays important roles in a number of intracellular signaling pathways and physiological functions that are regulated by neurotransmitters in the brain. Although more than 280,000 phosphorylation sites have been identified and are registered in databases such as PhosphoSitePlus (https://www.phosphosite.org/homeAction.action (accessed on 2 September 2022)), there are approximately 500 protein kinases encoded by the human genome, and the kinases that are directly responsible for these phosphorylation sites currently remain unknown. To overcome this issue, we developed the kinase-interacting substrate screening (KISS) method to isolate substrates by affinity purification with the responsible kinase and to analyze the substrates that interact with the kinase by liquid chromatography tandem mass spectrometry [97]. The KISS method was applied to identify the kinase substrates of nine different protein kinases, including PKA and MAPK1. Regarding the in vivo screening of kinase substrates, we developed the KiOSS method by using a wide range of reagents, such as phosphatase inhibitors, kinase inhibitors, kinase activators, cAMP phosphodiesterase inhibitors, and receptor agonists [98]. The KiOSS method facilitates the identification of the extracellular stimuli regulating protein phosphorylation via intracellular signaling cascades. As described above, we identified more than 100 novel PKA substrates downstream of dopamine/D1R signaling [42]. We recently reported data obtained on phosphoproteins and phosphorylation sites for adenosine/A2AR signaling and downstream MAPK-mediated signaling in the striatum/NAc [99]. To provide information on the phosphorylation signals identified by our KISS and KiOSS methods, as well as those previously reported in the literature, we developed a novel online database, KANPHOS (https://kanphos.neuroinf.jp (accessed on 6 September 2022)). The KANPHOS database allows users to not only search for substrates phosphorylated by a specific kinase, but to also search for kinases phosphorylating a specific protein or to search for kinases and their target substrates involved in a specific signaling pathway.

## 6. Actions of Dopaminergic and Adenosinergic Drugs in Neuropsychiatric Disorders

Dopamine precursors (e.g., levodopa), dopamine agonists (e.g., apomorphine), and drugs that block dopamine metabolism (e.g., monoamine oxidase type B inhibitors, catechol-O-methyl transferase inhibitors) are commonly prescribed to treat Parkinson’s disease [100,101]. These drugs lose efficacy when dopaminergic neurodegeneration progresses over time. The loss of dopaminergic neurons in the substantia nigra results in a reduction in or the loss of the excitatory effects of D1R and the inhibitory effects of D2R on the striatum (Figure 1c). A2A antagonists have recently been used in the symptomatic treatment of Parkinson’s disease [102,103], because the blockade of A2AR leads to motor activation by reducing D2R-MSN activity, similar to the activation of D2R [104]. Amphetamine and methylphenidate are first-line pharmacotherapies for ADHD, which are related to increasing dopamine activity in brain regions that include the cortex and striatum [105]. The role of dopamine receptors in ADHD has also been extensively examined [106]. D2R antagonists are commonly used as pharmacotherapy for positive symptoms in schizophrenia [107]. A recent study has also implicated the effect of antipsychotics on the D1R expression of monocyte-derived-neuronal-like cells in patients with schizophrenia [108]. The hypothesis that dopamine is involved in the causation of schizophrenia is partly derived from the mechanisms of action of antipsychotic drugs targeting dopamine receptors [109].

Although the locations of the target receptors of these drugs in the brain have been extensively examined, the underlying mechanisms and the sites at which these drugs act at the cellular and molecular levels remain largely unknown, which has been a major obstacle to the development of new therapeutic drugs. Recent advances in intracellular signaling analyses are expected to overcome these issues. For example, we demonstrated that a D1R agonist activated PKA and induced the phosphorylation of Rasgrp2/Rap1gap in D1R-MSN. A D2R antagonist activated PKA and induced Rasgrp2/Rap1gap phosphorylation in D2R-MSN. Furthermore, the D2R antagonist-induced activation of PKA and Rasgrp2/Rap1gap phosphorylation was suppressed by pretreatment with an A2AR antagonist [39,42]. The effects of D1R agonists, D2R antagonists, and A2AR antagonists may be examined at cellular and molecular levels by monitoring the phosphorylation of Rasgrp2/Rap1gap. Therefore, monitoring the phosphorylation of Rasgrp2/Rap1gap, which reflects the PKA activity, is important for examining the modes of action of existing drugs for neuropsychiatric disorders, and may also be useful for screening novel therapeutic agents in the development of new drugs.

## 7. Conclusions

As protein phosphorylation is critical for intracellular signaling pathways and physiological functions that are regulated by neurotransmitters in the brain, researchers have continued the search for kinase substrates in order to elucidate the molecular mechanisms underlying brain functions. Dr. Paul Greengard was the first to examine PKA substrates in various brain regions, and found that DARPP-32 amplified the effects of PKA downstream of dopamine, which gained him the Nobel Prize for Medicine in 2000. We recently focused on the screening of phosphorylation signals downstream of dopamine and shared the information obtained in the newly-established KANPHOS database, which may be freely accessed.

**Figure 3:**
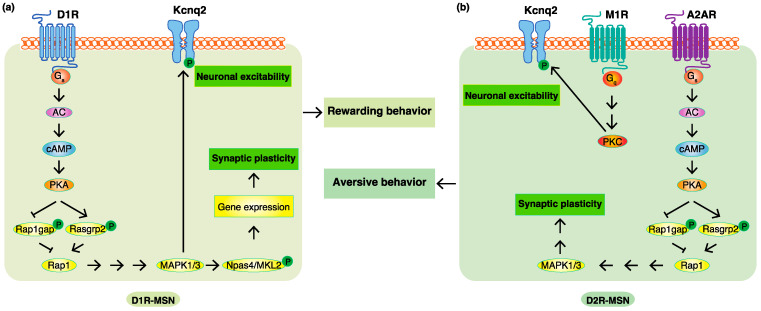
Phosphoproteomic analyses revealed new phosphorylation signals downstream of dopamine receptors in emotional behaviors. (**a**) In D1R-MSN, the stimulation of D1R by dopamine increased intracellular cAMP concentrations through AC, and this is followed by the activation of PKA. PKA phosphorylates Rasgrp2 and Rap1gap to induce the activation of Rap1, which promotes the MAPK pathway. MAPK phosphorylates Kcnq2 to increase membrane excitability and phosphorylates Npas4 and MKL2 to facilitate the expression of the genes accounting for synaptic plasticity, consequently leading to rewarding behavior. (**b**) In D2R-MSN, a decreased dopamine concentration cancels the suppressive effects of D2R. The stimulation of A2AR without the inhibition of D2R increases intracellular cAMP concentrations through AC, and this is followed by the activation of PKA. PKA phosphorylates Rasgrp2 and Rap1gap to induce the activation of Rap1, which promotes the MAPK pathway involved in synaptic plasticity. Acetylcholine/M1R signaling activates PKC to promote the phosphorylation of Kcnq2, which is involved in membrane excitability. These changes consequently lead to aversive behavior. D1R, dopamine D1 receptor; MSN, medium spiny neuron; Gs, stimulatory G protein; AC, adenylate cyclase; cAMP, cyclic adenosine monophosphate; PKA, protein kinase A; Rap1gap, rap1 GTPase-activating protein; Rasgrp2, Ras guanyl releasing protein 2; MAPK1/3, mitogen-activated protein kinase 1/3; Npas4, neuronal Per Arnt Sim domain protein 4; MKL2, megakaryoblastic leukemia-2; M1R, muscarinic M1 receptor; A2AR, adenosine A2A receptor; Gq, Gq protein; PKC, protein kinase C; Kcnq2, potassium voltage-gated channel subfamily Q member 2; P, Phosphate.

According to previous studies and our KANPHOS data, the stimulation of D1R by dopamine increases intracellular cAMP concentrations through AC, and this is followed by the activation of PKA. PKA phosphorylates Rasgrp2 and Rap1gap to induce the activation of Rap1, which promotes the MAPK pathway. MAPK phosphorylates Kcnq2 to increase membrane excitability. MAPK also phosphorylates Npas4 and MKL2 to facilitate the gene expression, accounting for synaptic plasticity regulated by dopamine. The stimulation of A2AR without the inhibition of D2R also increases intracellular cAMP concentrations through AC, and this is followed by the activation of PKA. The activation of Rap1 induces the MAPK pathway to regulate synaptic plasticity. The stimulation of M1R induces the activation of PKC, and this is followed by the phosphorylation of Kcnq2, which accounts for the increased membrane excitability. Therefore, phosphorylation signals downstream of D1R are involved in D1R-MSN-mediated rewarding behavior, and phosphorylation signals downstream of A2AR and M1R without the inhibition of D2R are involved in D2R-MSN-mediated aversive behavior (Figure 3).

Although we focused on conditioned place preference and passive avoidance behaviors as emotional behaviors in this review, there are other different rewarding and aversive stimuli (e.g., social reward/stress and alcohol) that affect intracellular signaling [110,111,112,113,114,115,116]. Emotional stimuli also lead to neuronal plasticity through protein expression. We believe that the mechanism through which emotional stimuli regulate brain function should be elucidated in a multi-scale manner from molecular to behavioral levels.

## Data Availability

The KANPHOS database may be accessed at the following website (https://kanphos.neuroinf.jp (accessed on 6 September 2022)).
